# One-month alcohol abstinence national campaigns: a scoping review of the harm reduction benefits

**DOI:** 10.1186/s12954-022-00603-x

**Published:** 2022-03-04

**Authors:** Julia de Ternay, Pierre Leblanc, Philippe Michel, Amine Benyamina, Mickael Naassila, Benjamin Rolland

**Affiliations:** 1 Service d’Addictologie, Hôpital Édouard Herriot, Hospices Civils de Lyon, 5, Place d’Arsonval, Pavillon K, 69003 Lyon, France; 2 Research on Healthcare Performance (RESHAPE), INSERM U1290, Hospices Civils de Lyon, Lyon, France; 3 Hôpital Paul Brousse, AHPH, Université Paris-Sud, Paris, France; 4 Unité Psychiatrie-Comorbidités-Addictions (PSYCOMADD), APHP, Université Paris-Sud, Paris, France; 5 Groupe de Recherche sur l’Alcool et les Pharmacodépendances (GRAP), INSERM U1247, Université de Picardie Jules Verne, Amiens, France; 6 Service Universitaire d’Addictologie de Lyon (SUAL), CH Le Vinatier, Bron, France; 7 CRNL PSYR2, Inserm U1028, CNRS UMR 5292, Université Claude Bernard Lyon 1, Bron, France

**Keywords:** Dry January, One-month abstinence, Alcohol abstinence, Abstinence campaigns

## Abstract

Over the last decade, one-month alcohol abstinence campaigns (OMACs) have been implemented within the general population in an increasing number of countries. We identified the published studies reporting data on OMACs to explore the following aspects: profile of participants, rates and factors associated with the completion of the abstinence challenge, and outcomes and harm reduction benefits in participating in the challenges. We screened 322 records, including those found in the grey literature, and reviewed 6 studies and 7 Dry July Annual Reports. Compared to non-participating alcohol users, participants were more likely to be female, have a higher income, and a higher level of education. They were heavier drinkers and were more concerned by the consequences of alcohol on health and by their health in general. Participants who achieved the one-month abstinence challenge were lower drinkers and more likely to have registered on the campaign-related Internet communities. Both successful and unsuccessful participants frequently reported health benefits, including sleep improvement and weight loss. Successful participants were more likely to durably change their alcohol drinking habits. Overall, OMACs provide short- or mid-term harm reduction benefits for both successful and unsuccessful participants. Findings were limited by the paucity of studies, their observational nature, and heterogeneity in the features of the different national campaigns, which would probably gain in enhanced internationalization.

## Introduction

Alcohol consumption is ingrained in the cultural habits of many countries, in particular in “Western” countries. However, an accumulating body of evidence indicates that both the amount and frequency of alcohol use are directly associated with an increased mortality resulting from various medical risks, including cancer [1], alcohol-related liver diseases [2], stroke, coronary disease, heart failure, hypertensive disease, and aortic aneurysm [3]. Public health strategies thus aim to promote an overall reduction in alcohol use among the general population.

In this respect, public health campaigns, challenging the general public to temporarily stop alcohol consumption, have been spreading over recent years, as it has also been the case regarding tobacco consumption with the emergence of national and international contests for smoking cessation since the 1980s [4], and of national campaigns, such as the “Stoptober” in the UK since 2012 that promotes a cessation of tobacco smoking for at least 28 days [5]. Temporary alcohol abstinence campaigns aim to promote behavioral changes and general health improvements among participants, which can involve many dimensions of health, including improved sleep, weight loss, increased physical activity, or enhanced quality of life. As such, adding to these elements the fact that these campaigns do not necessarily promote a complete cessation of alcohol consumption in the long term, but rather encourage drinkers to question their relationship with alcohol and its consequences on their health, they can be conceived as harm reduction programs applied to the general population. In practice, however, temporary abstinence campaigns have generally been implemented at a national level, and the way they have been set up may thus largely differ between countries. In particular, the time frame of the abstinence period challenge can be very variable, depending on the program. In some programs, this abstinence period is quite long, e.g., three months in the Buddhist Lent Dry Campaign abstinence in Thailand [6], or even twelve months [7]. However, the longer the targeted abstinence period is, the more the participants’ characteristics may differ from the general population participating in shorter abstinence periods. For example, 95% of the people participating in the Australian Hello Sunday Morning (HSM) program, an online Australian program promoting alcohol abstinence for three or twelve months, reported a harmful alcohol use before engaging in the program [7].

By contrast, the most widespread prevention initiatives that promote temporary alcohol cessation within the general population consist of one-month-long abstinence campaigns (OMACs). This is the case of one of the most popular of these programs, the Dry January challenge, launched in 2013 in the UK (“Dry January | Alcohol Change UK”) [8]. Similar January OMACs now exist in Quebec (“Défi 28 jours sans alcool”), France (“Dry January | Le défi de janvier !”), and in the Netherlands (“IkPas”) [9], while other months of the year have been chosen in other countries, i.e., February in Belgium (“Tournée Minérale—Een maand zonder alcohol”) [10] and New-Zealand from 2011 to 2015 (“FebFast NZ | NZ Drug Foundation”), and November in Hungary (“Száraz November”) [11]. In Australia, three campaigns have co-existed for ten years, the first, Dry July, started in 2008 and still exists (“Go Dry this July”) [12] while the second, Febfast was established in 2007 in Australia and in 2011 in New Zealand [14] and the third, Ocsober, was discontinued in 2019 after having been running for ten years (“Life Education”) [13]. Another common feature of OMACs is that participants can sign up on social media, and thus receive and post-supportive messages, which makes them belong to online communities that may foster their personal efforts [15]. Other inconstant features are the fundraising aspect of some campaigns (in particular in the Australian programs) where the participants can buy one-day leave passes if they wish to withdraw from the program only for one day.

In this review, we addressed the harm reduction benefits of OMACs, that is, the features of participants, the rates and predictors of success, i.e., completing the abstinence challenge, and the health benefits reported by participants, including those who did not fulfill the challenge. The aim of this review was to provide a state-of-the-art of the demonstrated evidence regarding the harm reduction benefits of OMACs, but also to determine which additional research questions should be addressed in the upcoming years, and which populations should be more specifically targeted in future OMACs, in particular those who could currently not been reached by the existing programs.

## Methods

We conducted a systematic search in PubMed, ScienceDirect and PsycInfo from inception to 30/08/2021 using the following keyword algorithm: *(“Dry January” OR “Dry July” OR “Dry November” OR “Ocsober” OR “IkPas” OR “Febfast” OR “la Tournée minerale” OR “défi 28 jours” OR “temporary abstinence”) AND alcohol*. The title and abstract of the records identified through the search algorithm were independently reviewed by two authors (JdT and PL). Disagreements were resolved by two senior authors (BR and MN). Additional records could be added using the reference list of the included articles and the grey literature (World Health Organization database, Virtual health database, OpenGrey database, available reports from websites of OMACs).

The studies included for review had to be written in English, have a quantitative research method, and report data on one of the following items: (1) the characteristics of individuals participating in the program(s), (2) the proportion of participants who reached the target of one-month abstinence, (3) the individual predictive factors of success or failure for completing the OMAC, and (4) the outcomes reported by the participants. We chose to focus only on epidemiological studies providing quantitative data and pertaining to the general population, studies with a local or regional focus were therefore excluded, as were those with experimental research design, studies focusing on alcohol abstinence campaigns longer than one-month, qualitative studies, as well as letters or articles providing no original data.

For each study, the following data were extracted in calibrated form: authors’ name, publication year, country, publication type, study methodology, name of the abstinence challenge and year of the event, main results and others findings. The characteristics of each study were extracted by two authors (JdT and PL), and disagreements were resolved by two senior authors (BR and MN). For each study, the descriptive results corresponding to the objectives of the review (see above) were reported into the Results section. All authors participated in the appraisal and synthesis of the results.

## Results

**Fig. 1:**
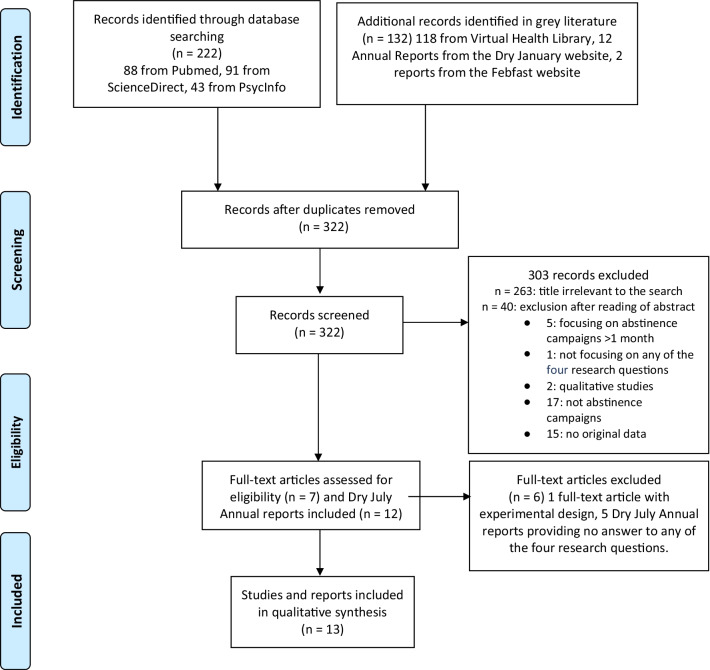
Flowchart of reviewed studies from literature search to inclusion in the scoping review

The database search identified 222 records (PubMed, ScienceDirect, PsycInfo), and 132 records were identified in grey literature. Thirty-two duplicates were removed. The title and abstract of 322 individual records were screened, 309 of which were irrelevant and excluded. The 19 remaining records included seven studies and the 12 Dry July Annual reports that underwent full-text examination. One of the studies was then excluded because of its experimental research design [16] as well as five Dry July Annual reports providing no answer to any of the four research questions. Six studies [17–22] and seven Dry July Annual reports [23–29] were finally included for the review (Fig. 1). The six studies were published between 2016 and 2021, five of them referred to Dry January [17–20, 22], and one pertained to Febfast 2012 [21]. Characteristics of the reviewed studies and reports can be found in Table 1.

**Table 1:** Characteristics of the reviewed studies

Author(s). Year, *Country*	Challenge(s)	Objective(s)	Study type/design	Population/sample size (*n*)	Data collection and analysis/measurements
Thomson 2012 [21] *Australia*	Febfast	To understand: 1. Who took part in febfast and their reasons for participation 2. The experience of participating in febfast 3. The impact that taking part in febfast has on participants’ alcohol awareness, health and subsequent drinking behaviors 4. Awareness of febfast and barriers to participation among a sample of Australian drinkers	Quantitative/longitudinal study	1. Registrants to Febfast 2011/*n* = 1,330 2. Australian drinkers/*n* = 2,015	Febfast registrants: two online surveys, one during febfast 2011 and the other between June and July 2011, Australian drinkers: one survey/Both febfast participants and Australian drinkers: Demographics, alcohol consumption patterns, beliefs around the health risks and benefits risks associated with alcohol consumption, previous participation in febfast, awareness of other people taking part, knowledge of and participant in other similar programs (Hello Sunday Morning, Dry July, Ocsober), likelihood of future participation Febfast participants: smoking frequency during and after febfast, how they found out about the program, motivation for taking part, perceptions of febfast participation, use of "Time Out certificates", awareness of promotion of the Febfast event Australian Drinkers: smoking frequency, awareness of febfast, barriers participating in Febfast, Support for funds raised by febfast participants going to other charities (e.g., charities supporting cancers)
de Visser et al., 2016. [20] *UK*	Dry January	1. To determine what are Dry January predictors of success 2. To attest that successful completion of Dry January challenge leads to increase in DRSE and thus to a decrease in alcohol consumption 3. To assess the risk of rebound effect according to success or failure in the challenge	Quantitative/prospective cohort study	British adult participants having registered to Dry January 2014, who completed all three questionnaires/*n* = 857	Three visits: baseline, 1-month follow-up, 6-month follow-up/Demographic data, AUDIT* score, DRSE*** scale, age at first drink, longest previous abstinence period, Dry January companion (yes/no), fundraising during Dry January (yes/no), plan to stop drinking (yes/no), period between registration for the challenge and first alcohol drink (in days)
de Visser et al., 2017. [17] *UK*	Dry January	1. To explore the ‘diffusion’ of Dry January 2. To assess the benefits of official registration to Dry January compared to unofficial participation	Quantitative/cross-sectional study and Prospective cohort study	1. Adult drinkers in the general population/*n* = 825 in February 2015 and *n* = 874 in February 2016 2. Registrants for Dry January 2015/*n* = 13,277 3. Unofficial participants to Dry January/*n* = 1,251	Interviews conducted in February 2015 and 2016, three questionnaires: baseline, 1-month follow-up, at 6-month follow-up, data extraction: number of Dry January registrations between 2013 and 2016/AUDIT score, DRSE score, questions aiming to assess the awareness of Dry January in general population
de Visser and Nicholls, 2020. [18] *UK*	Dry January	1. To examine how successful and unsuccessful attempts at Dry January are related to benefits and overall and specific aspects of well-being) 2. To explore how successful or failed attempts at Dry January influence changes in GSE 3. To determine the perception and the official email support importance compared to other variables known to be associated with success to the Dry January	Quantitative/prospective cohort study	Registrants to Dry January 2016/*n* = 7,642 (including the 4,232 respondents to the two follow-up questionnaires)	Two questionnaires: at baseline, at the end of the challenge (February the 1st)/Background demographic data, 10-item AUDIT score, DRSE scale, GSE† scale, WEMWBS†† scale, previous participation to Dry January (yes/no), existence of a companion for Dry January (yes/no), delay between beginning of Dry January and first alcohol consumption (in days), 7-point scale to assess 5 domains of experienced benefits during the challenge, use of support emails (yes/no), 5-point scale about support use frequency and perceived efficacy
de Visser and Piper, 2020. [19] *UK*	Dry January	1. To determine the profile of Dry January participants compared to the rest of the population 2. To determine whether beneficial changes observed among successful Dry January participants are unique to that group or are also observed among unsuccessful participants and drinkers in the general population not trying to abstain from alcohol	Quantitative/prospective cohort-study	Official registrants to Dry January 2016 and adult drinkers from the general population/*n* = 3,171 registrants at baseline (1,342 at 1-month follow-up, 1,158 at 6-month follow-up) and *n* = 2,977 adult drinkers at baseline (2,222 at 1-month follow-up, 1,583 at 6-month follow-up). 34 adult drinkers at baseline recoded as Dry January registrants	Three online questionnaires: at baseline, at 1-month follow-up, at 6-month follow-up/Background demographic data, 10-point scale to self-report concern about drinking effect on health and control over drinking, self-rated physical well-being (5-point Likert-scale), 7-item WEMWBS scale, DRSE scale, AUDIT-C** score
Case et al. 2021. [22] *UK*	Dry January	To assess whether the increase in participation in Dry January between 2015 and 2018 was associated with reduced alcohol consumption in England, independently of pre-existing seasonal variation	Repeat cross-sectional design	Respondents to the Alcohol Toolkit Study aged 16 + years in England/*n* = 37,142	Data collected from March 2014 to January 2015 and March 2017 to January 2018, February excluded for both periods 1. Percentage of adults reporting drinking monthly or less frequently in the last 6 months (AUDIT-C) 2. Mean weekly alcohol consumption among drinkers, frequency and quantity in the last 6 months (AUDIT-C) 3. Percentage of at-risk drinkers reporting a current attempt to restrict alcohol consumption assessed by the question *"Are you currently trying to restrict your alcohol consumption (Yes/No)"* 4. Percentage of at-risk drinkers citing Detox/Dry January as a motive in their most recent attempt to restrict alcohol consumption assessed by the question *"Which of the following, if any, do you think contributed to making the most recent attempt to restrict your alcohol consumption?"* 5. Percentage of at-risk drinkers reporting use of a website or app to help to restrict alcohol consumption in their most recent attempt assessed with the question "*Which, if any, of the following did you use to try to help restrict your alcohol consumption during the most recent attempt?"* with the option: *"visited a website for help with drinking", "use an alcohol app' on a handheld computer"*
Dry July Annual Reports [23–29] *Australia* 2009/2010, 2010/2011, 2011/2012, 2012/2013, 2013/2014, 2019, 2020	Dry July	–	–	2019: Participants to Dry July *n* = 5,049	–

*AUDIT = Alcohol Use Disorders Identification Test (10-item scale), **AUDIT-C = Alcohol Use Disorders Identification Test-Consumption (3-item scale), ***DRSE =  Drink Refusal Self-Efficacy (7-point Likert scale with 9 items), ^†^GSE = General Self-Efficacy (4-point Likert scale with 10 items), ^††^WEMWBS = Warwick–Edinburgh Mental Well-Being Scale (5-point Likert scale with 7 or 14 items)

### Characteristics of individuals participating in these programs

Subjects aged between 25 and 35 years represented the highest proportion of participants in the Dry July campaign in 2010 (38.5%), 2011 (34.0%) and 2014 (38.0%) [23, 24, 27], respectively. Compared to a control group of non-participating alcohol users, registrants to Dry January and Febfast were found to be more likely female (75.3%, 95%CI [72.5; 77.8], vs. 50.9%, 95%CI [47.9; 53.9]; *p* < 0.01) [19, 21], be working [21], and to have completed university education, (48.1%, 95%CI [45.0; 51.2], vs. 37.7%, 95%CI [34.8; 40.6]; *p* < 0.01) [19, 21]. Registrants also had a higher income [21], a better self-rated physical health, a lower score on the Warwick–Edinburgh Mental Well-being Scale (3.37, 95%CI [3.32; 3.41] vs. 3.46, 95%CI [3.41; 3.52], *p* < 0.01) [30], and they were more concerned about the health consequences of drinking, and about their control over drinking [19]. They were more likely to agree that alcohol is a serious issue for the community, and less likely to believe that there were benefits associated with drinking alcohol [21]. They were also more likely to classify themselves as heavier drinkers [21] and had a higher score on the Alcohol-Use Disorders Identification Test—Consumption (AUDIT-C) [31] (8.47, 95%CI [8.27; 8.66] vs. 5.74, 95%CI [5.49; 6.00], *p* < 0.01). They exhibited a lower drink refusal self-efficacy score (DRSE), which is the perceived ability to refuse alcohol in different contexts—emotional, social and opportunistic [32] (4.30, 95%CI [4.21; 4.40] vs. 5.28, 95%CI [5.17; 5.39]; *p* < 0.01). During Dry January, they were more likely to have tried to increase their physical activity (48.7% vs. 23.8%; *p* < 0.01) or to improve their diet (52.3% vs. 28.2%; *p* < 0.01) [19] (Table 2).

**Table 2:** Results extracted for each research question

Research questions	(1) What were the characteristics of individuals participating in these programs?	(2) Which proportion of participants could reach the 1-month abstinence?	(3) What were the individual predictive factors of success or failure for completing the 1-month abstinence challenge?	(4) What were the outcomes reported by the participants?	Other findings
Thomson 2012 [21]	Febfast registrants: more likely to be females, be aged between 25 and 54, reside in Victoria, have a higher household income, be working, have completed university (*p* < 0.001), have been born in Australia, be living in a nuclear family or share house (*p* < 0.001), agree that alcohol is a serious issue for the community (*p* < 0.001), classify themselves on the heavier spectrum of drinking style (*p* < 0.001). Less likely to refuse alcohol when offered to them (*p* < 0.001), believe that there were benefits (*p* < 0.001) associated with drinking alcohol. 75.9% of the participants participating for the first time in 2011, those aged 45–54 and 55 + more likely to have participated more than once (*p* < 0.01). Specific motivations: registrants wanting to “give their body a break” more likely to be aged 25–44 (*p* < 0.05), have a higher income (*p* < 0.05), have completed febfast more than once (*p* < 0.05), be heavier drinkers (*p* < 0.001). Respondents motivated by “getting out of drinking habits” more likely to have a higher income (*p* < 0.05), be aged 35–54 (*p* < 0.05), classify themselves as heavier drinkers (*p* < 0.01). Motivation to participate to lose weight or to improve health more likely associated with being a heavier drinker (*p* < 0.001). 81.1% reported knowing at least one person who had also participated (*p* < 0.001)	–	Heavier drinkers more likely not to complete the event (*p* < 0.01)	25% reporting giving up alcohol for a month was difficult or very difficult. Younger participants more likely to find the experience difficult compared to those aged 45 or older, as those with heavier drinking patterns (*p* < 0.001).46.5% reporting reduced amount of tobacco products consumed during febfast. 14.7% having learnt new information through febfast (health risk associated with alcohol for 32.0%). 85% of the participants reporting at least one benefit. Most commonly reported benefits: saving money (52.2%), improved sleep (40.5%), weight loss (38.1%), improved overall health (35.3%)	Febfast registrants participating the survey: more likely to be females (74.5% vs. 62.0% of the 2011 participants), to have participated more than once Febfast respondents: 92.7% inclined to recommend the event to others. 68.4% intending to participate to febfast 2012. 25% purchased at least one Time Out Certificate. Females, participants aged 25–44, those without previous participation to febfast (*p* < 0.05), heavier drinkers (*p* < 0.01) more likely to purchase certificates. 45.2% having knowledge of ‘unofficial participants’ doing febfast. Among those having reduced their consumption during febfast, 68.8% reporting maintaining the change after February (21.2% planning to maintain reduced consumption because of health) Reduced frequency of alcohol consumed following febfast: associated with having a higher income and lighter current drinking patterns (*p* < 0.05), having experienced benefits while completing febfast (*p* < 0.001), with each individual benefit significantly associated with a reduced frequency (*p* < 0.01), except being motivated to save money. Those motivated by the desire to break their drinking habits, to give their body a break from alcohol, improve health, save money, lose weight, or for the personal challenge more likely to reduce frequency of consumption (*p* < 0.05) Reduced amount of alcohol consumed following febfast: associated with having experienced benefits while completing febfast (*p* < 0.001), being motivated to break drinking habits, give the body a break, improve health, lose weight, or seeing febfast as a personal challenge. Motivation to participate with others associated with a decreased likelihood of reducing the amount consumed (*p* < 0.01) Australian drinkers’ sample: Less than 30% somewhat or very likely to participate to febfast in the future
de Visser et al. 2016 [20]		64.1% of successful respondents	One independent predictor of success: lower frequency of drunkenness at baseline OR = 0.93, 95%CI [0.90; 0.96]. Correlates of success (at baseline): fewer drinks per drinking days (3.78 vs. 4.21, d* = 0.21, *p* = 0.01), lower frequency of drunkenness the month before (2.55 vs. 3.84, *d* = 0.36, *p* < 0.01), lower AUDIT-score (11.09 vs. 12.56, *d* = 0.26, *p* < 0.01), Greater social (3.61 vs. 3.23, *d* = 0.23, *p* < 0.01) and emotional (4.35 vs. 4.05, *d* = 0.16, *p* = 0.02) DRSE scores	Risk of rebound effect: 11% of reported increased frequency of drunkenness at 6-month follow-up. Unsuccessful participants more likely to report an increase in frequency of drunkenness at 6-month follow-up (*p* < 0.01, *d* = 0.39). Successful participants: Increase in all 3 dimensions of the DRSE score at 1-month follow-up (social *p* < 0.01, *d* = 0.39, emotional *p* < 0.01, *d* = 0.30, opportunistic *p* < 0.01, *d* = 0.23). Reduction in alcohol intake at 6-month follow-up (drinking days per week *p* < 0.01, *d* = 0.53, drinks per drinking day *p* < 0.01, *d* = 0.25, drunk episodes last month *p* < 0.01, *d* = 0.39). Unsuccessful participants: increase in DRSE social (*d* = 0.11, *p* = 0.03), DRSE emotional (*d* = 0.23, *p* < 0.01) at 1-month follow-up, reduction in drinking days per week (*d* = 0.45, *p* < 0.01), drinks per typical drinking day (*d* = 0.18, *p* < 0.01) and frequency of drunkenness at 6-month follow-up (*d* = 0.39, *p* < 0.01)	People who completed all three questionnaires: older, more likely to have completed a dry month in the past, fewer drinks per drinking day, less frequent drunkenness, lower AUDIT score, and greater social DRSE. Successful participants: Similar proportion of males and females (female 62.9%, male 66.7%, *p* = 0.29)
de Visser et al. 2017 [17]	–	62% of successful registrants	–	Registrants: at 6-month follow-up lower AUDIT scores (*p* < 0.01), elevated DRSE scores (social DRSE *p* < 0.01, emotional DRSE *p* < 0.01, opportunistic *p* < 0.01) compared to the unofficial participants	15-fold increase in participation between 2013 (4,000) and 2016 (60,000). Unofficial participants: 7% tried not to drink in January in 2015, and 11% in 2016. Adult drinkers: 64% aware of Dry January in 2015 and 78% in 2016. Successful participants: 96% reported signing up to receive official supportive emails, 69% read every message sent, 71% considered the messages helped them succeed. Successful and unsuccessful participants: 57% chose to receive supportive messages, 78% considered that helped them, 42% used social media support and 73% considered such support helped them
de Visser and Nicholls 2020 [18]	–	61% of successful respondents	Four independent predictors of success: being male rather than female (OR = 1.46, 95%CI [1.35; 1.58]), having a lower AUDIT score (OR = 0.97, 95%CI [0.96; 0.98]) or a greater emotional DRSE score at baseline (OR = 1.09, 95%CI [1.06; 1.11]), reading supportive emails ‘always’ rather than ‘never’ (OR = 1.81, 95%CI [1.65; 1.97]). Correlates of successful completion of Dry January: being a male (66.4% vs. 59.2% for females, *V* = 0.05, *p* < 0.01), lower AUDIT score (*d* = 0.24, *p* < 0.01), lower frequency of drunkenness (*d* = 0.19, *p* < 0.01), higher DRSE social (*d* = 0.21, *p* < 0.01), emotional (*d* = 0.23, *p* < 0.01) and opportunistic (*d* = 0.18, *p* < 0.01) scores, higher GSE score (*d* = 0.10, *p* < 0.01), higher WEMWBS score (*d* = 0.12, *p* < 0.01), higher frequency of reading support emails (*V* = 0.08, *p* < 0.01)	All Participants: increased WEMWBS scores (*d* = 0.34, *p* < 0.01) and the GSE scores (*d* = 0.12, *p* < 0.01) at 1-month follow-up, savings (63%), improved sleep (56%), increased energy (52%), greater health (50%), weight loss (38%). Positive effects of Dry January on savings, sleep, energy, health and weight loss found more frequently in the successful participants' group (savings *d* = 0.13, *p* < 0.01; sleep *d* = 0.15, *p* < 0.01; energy *d* = 0.14, *p* < 0.01; health *d* = 0.12, *p* < 0.01; weight *d* = 0.24, *p* < 0.01) Successful participants: Increase in GSE score (*d* = 0.12, *p* < 0.01) at 1-month follow-up. Unsuccessful participants: no significant change in GSE scores	–
de Visser and Piper 2020 [19]	Participants: more likely to be female (75.3% vs. 50.9%, *p* < 0.01), younger (45.41 vs. 49.82, *p* < 0.01), higher mean income (*p* < 0.01), more likely to have completed university education (48.1% vs. 37.7%, *p* < 0.01), better self-rated physical health (3.23 vs. 2.93, *p* < 0.01), lower WEMWBS scores (3.37 vs. 3.46, *p* < 0.01), more concern about the health effects of drinking (6.60 vs. 4.47, *p* < 0.01) and about control over the drinking (5.53 vs. 3.72, *p* < 0.01), higher AUDIT-C scores (8.47 vs. 5.74, *p* < 0.01), lower DRSE (4.30 vs. 5.28, *p* < 0.01). Significantly more likely to have tried to engage in more physical activity (48.7% vs. 23.8%, *p* < 0.01) or to improve their diet (52.3% vs. 28.2%, *p* < 0.01)	62.4% of successful respondents	–	Successful participants: increase in self-rated physical-health (baseline mean = 3.26, 95%CI [3.17; 3.35], 1-month follow-up mean = 3.47, 95%CI [3.39; 3.46], 6-month follow-up mean = 3.47, 95%CI [3.39; 3.56]), DRSE scores (baseline mean = 4.27, 95%CI [4.14; 4.40], 1-month follow-up mean = 4.86, 95%CI [4.73; 4.98], 6-month follow-up mean = 4.83, 95%CI [4.69; 4.96]), WEMWBS scores (baseline mean = 3.40, 95%CI [3.34; 3.47], 1-month follow-up mean = 3.77, 95%CI [3.71; 3.83], 6-month follow-up mean = 3.68, 95%CI [3.62; 3.74]), decrease in AUDIT-C scores (baseline mean = 8.89, 95%CI [8.65; 9.12], 6-month follow-up mean = 6.72, 95%CI [6.37; 7.07]). Unsuccessful participants: increase in self-rated physical-health (baseline mean = 3.12, 95%CI [3.01; 3.24], 1-month follow-up mean = 3.20, 95%CI [3.07; 3.32]), 6-month follow-up mean = 3.16, 95%CI [3.04; 3.28]), DRSE scores (baseline mean = 4.63, 95%CI [4.45; 4.82], 1-month follow-up mean = 5.04, 95%CI [4.88; 5.21], 6-month follow-up mean = 4.94, 95%CI [4.76; 5.12]), WEMWBS scores (baseline mean = 3.37, 95%CI [3.28; 3.47], 1-month follow-up mean = 3.56, 95%CI [3.48; 3.66], 6-month follow-up mean = 3.49, 95%CI [3.39; 3.58]), decrease in AUDIT-C scores (baseline mean = 6.82, 95%CI [6.37; 7.27], 6-month follow-up mean = 6.18, 95%CI [5.76; 6.59])	Official registrants: more likely to complete the challenge (69.8% vs. 30.2%, *p* < 0.01) Unofficial participants: 30.2% Dry January completion (*p* < 0.01)
Case et al. 2021 [22]	–	–	–	(1) Percentage of adults reporting drinking monthly or less frequently: lower in January than non-January months both in 2014/2015 (46% vs. 49%) and 2017/2018 (45% vs. 51%), (2) Mean weekly alcohol consumption among drinkers: no significant differences between January and non-January months (β** = 0.23, 95%CI [− 0.11;0.58]) (3) Percentage of at-risk drinkers reporting a current attempt to restrict alcohol consumption: higher in January than non-January months in both 2014/15 (25% vs. 20%) and 2017/18 (27% vs. 19%). Odds of at-risk drinkers reporting current attempt to restrict consumption significantly higher in January vs. non-January (OR = 1.46, 95%CI [1.25;1.70]) (4) Percentage of at-risk drinkers citing Detox/Dry January as a motive in their most recent attempt to restrict alcohol consumption: higher in January than non-January months in both 2014/15 (13% vs. 4%) and 2017/18 (18% vs. 11%). Odds of at-risk drinkers citing Dry January as a motive in their most recent attempt to restrict consumption significantly higher in January vs. non-January (OR = 2.29, 95%CI [1.62;3.22]) (5) Percentage of at-risk drinkers reporting use of a website or app to help restrict alcohol consumption in their most recent attempt: No significant differences in January vs. non-January (OR = 0.77, 95%CI [0.33;1.78]), same percentage in January vs. non- January months in 2014/15 (2%) and similar in January vs. non-January in 2017/18 (2% vs. 3%)	(1) Percentage of adults reporting drinking monthly or less frequently: Odds of reporting drinking monthly or less frequently significantly higher in 2017/2018 vs. 2014/2015 (OR = 1.06, 95%CI [1.02;1.10]). Odds of reporting drinking monthly or less frequently in January vs. non-January months not significantly different between 2017/18 and 2014/15. Differences between January and other months similar in 2014/15 and 2017/18 for adults reporting drinking monthly or less frequently and mean consumption among drinkers (OR = 0.91, 95%CI [0.79;1.05], BF*** = 0.05; *β* = 0.55, 95%CI [− 0.14;1.25], BF = 0.13 , respectively) (2) Mean weekly alcohol consumption among drinkers: No significant differences between 2017/18 and 2014/15 (*β* = − 0.003, 95%CI [− 0.20;0.20]) (3) Percentage of at-risk drinkers reporting a current attempt to restrict alcohol consumption: No significant difference between 2017/18 and 2014/15 (OR = 0.92, 95%CI [0.84;1.02] (4) Percentage of at-risk drinkers citing Detox/Dry January as a motive in their most recent attempt to restrict alcohol consumption: Odds of citing Dry January significantly higher in January vs. non-January in 2014/15 (OR = 3.86, 95%CI [2.15;6.92]) vs. 2017/18 (OR = 1.81, 95%CI [1.18;2.79]). Odds of risky drinkers citing Dry January as a motive in their most recent attempt to restrict alcohol consumption significantly higher in 2017/18 vs. 2014/15 (OR = 2.59, 95%CI [1.90;3.53]) (5) Percentage of at-risk drinkers reporting use of a website or app to help to restrict alcohol consumption in their most recent attempt: No significant differences between 2017/18 and 2014/15 (OR = 1.57, 95%CI [0.94;2.61]). Respondents: Mean weekly consumption ranged from 5.2 units (SD: 8.8) (January 2015) to 5.7 units (SD: 10.3) (January 2018). Of the total sample, 26% were at-risk drinkers (scoring ≥ 5 on AUDIT-C)
Dry July Annual Reports [23–29] 2009/2010, 2010/2011, 2011/2012, 2012/2013, 2013/2014, 2019, 2020	2009/2010: 43% males, 57% females, 12.9% aged 18–25 years old, 38.5% aged 25–35 years old, 25.0% aged 35–45 years old, 15.2% aged 45–55 years old, 6.0% aged 55 years old or more. 2010/2011: 46% males, 54% females, 15.3% aged 18–25 years old, 34.0% aged 25–35 years old, 23.9% aged 35–45 years old, 17.0% aged 45–55 years old, 9.8% aged 55 years old or more. 2011/2012: 43% males, 57% females, 2012/2013: 43% males, 57% females. 2013/2014: 43% males, 57% females, 14% aged 18–24 years old, 38% aged 25–34 years old, 24% aged 35–44 years old, 14% aged 45–54 years old, 10% aged 55 years old or more. Motivations reported for taking part in the challenge: 59% due to friends or family being affected by cancer, 45% to see if able to complete the challenge, 44% to support their local hospital	–	–	2012 Participants: 76% willing to drink less having completed Dry January, 42% having changed their drinking habits post-Dry July. Mid-year health check: 36% having changed their diet, 36% having increased their current exercise program, 33% going to the gym as an alternative to drinking, 34% visiting their family/friends just because of not drinking. 2013 Participants: 74% willing to drink less having completed Dry January, 44% having changed their drinking habits post-Dry July. Mid-year health check: 22% having changed their diet, 22% having increased their current exercise program, 20% going to the gym as an alternative to drinking 2019 Participants: 21% feeling healthier. 2020: 98% reporting a positive experience	2010/2011: 99% reporting Dry July to be a positive experience, 98% planning to participate again. 2011/2012: 99% reporting Dry July to be a positive experience, 53% reporting that the most rewarding part was sense of achievement, 97% intending to recommend Dry July to friends, family and work colleagues. 2012/2013: 99% reporting Dry July to be a positive experience, 36% reporting that the most rewarding part was sense of achievement, 73% intending to participate to Dry July 2014, 96% intending to recommend Dry July to friends, family and work colleagues. 2019: 42% reporting sense of achievement 2020: 75% intending to do dry again to support the cause, 45% reporting the sense of achievement to be the most rewarding

*Effect size (Cohen's d, Cramer's V)

**Standard regression coefficient *β*

***Bayes factor

### Success rates

Between 61 and 64% [17–20] of registrants reported they had successfully completed Dry January whereas only 30.2% of the unofficial participants, i.e., participants who had not officially registered on the Dry January application or website, declared having completed it (*p* < 0.01) [19] (Table 2).

### Predictive factors of success or failure

Studies on Dry January found that participants were more likely to complete the program if they had fewer drinks per drinking day (*d* = 0.21; *p* = 0.01), lower frequency of drunkenness (*d* = 0.19–0.36; *p* < 0.01), a lower AUDIT score (*d* = 0.26; *p* < 0.01, 9.80 vs. 11.48, *d* = 0.24; *p* < 0.01), higher social (*d* = 0.21–0.23; *p* < 0.01), emotional (*d* = 0.16; *p* = 0.02, *d* = 0.23; *p* < 0.01) and opportunistic DRSE scores (*d* = 0.18; *p* < 0.01), greater mental well-being (*d* = 0.12; *p* < 0.01), higher general self-efficacy score (i.e., the perceived self-efficacy of being able to adapt and cope with various situations) (*d* = 0.10; *p* < 0.01) at baseline, and had read all the supportive emails during the campaign (*V* = 0.08; *p* < 0.01) [18, 20]. Heavier drinkers were more likely to fail to complete the event during Febfast [21]. A similar proportion of males and females reported success (females 62.9%, males 66.7%, *p* = 0.29) [20] (Table 2), but in another study, after adjustment, being a male was an independent predictor of a successful challenge (OR = 1.46, 95%CI [1.35; 1.58]) [18].

### Outcomes reported by participants

Twenty-five percent of the Febfast registrants reported giving up alcohol for a month was difficult or very difficult, especially younger participants and participants with heavier drinking patterns [21]. Dry January registrants had a higher DRSE score at 1-month and 6-month follow-up when they were successful (baseline: 4.27, 95%CI [4.14; 4.40], 1-month follow-up: 4.86, 95%CI [4.73; 4.98], 6-month follow-up: 4.83, 95%CI [4.69; 4.96]) [19]. This was also the case for those who were unsuccessful at 1 month (baseline: 4.63, 95%CI [4.45; 4.82], 1-month follow-up: 5.04, 95%CI [4.88; 5.21]), and there was a trend toward this at 6 months (4.94, 95%CI [4.76; 5.12]) [33]. The successful participants’ DRSE score increased in all three dimensions, whereas only the social and emotional dimensions increased for the unsuccessful participants [20]. No significant change in DRSE was found among unofficial participants [17].

Among the successful registrants, there was a decrease in the AUDIT-C score from baseline (8.89, 95%CI [8.65; 9.12]), to the 6-month assessment (6.72, 95%CI [6.32; 7.07]) [19], as well as significant reductions in the number of drinking days per week (*d* = 0.53; *p* < 0.01), number of drinks per drinking day (*d* = 0.25; *p* < 0.01), frequency of drunkenness (*d* = 0.40; *p* < 0.01) at 6-month follow-up [20]. The same improvements were found among unsuccessful registrants (AUDIT-C at baseline: 6.82, 95%CI [6.37; 7.27], at 6-month follow-up: 6.18, 95%CI [5.76; 6.59], drinking days per week *d* = 0.45; *p* < 0.01, drinks per drinking day *d* = 0.18; *p* < 0.01, frequency of drunkenness *d* = 0.39; *p* < 0.01) [19, 20]. However, unsuccessful registrants were more likely to report an increased frequency of drunkenness after Dry January compared to those who were successful (14.6% vs. 8%; *p* < 0.01) [20].

Only successful Dry January registrants had a higher general self-efficacy score at 1-month follow-up (3.19 at baseline vs. 3.25 at follow-up, *p* < 0.01) [18].

The Warwick–Edinburgh Mental Well-being Scale score was higher at 1-month follow-up for all participants (*d* = 0.34, *p* < 0.01) [18] and 6-month follow-up for both successful (baseline: 3.40, 95%CI [3.34; 3.47], follow-up: 3.68, 95%CI [3.62; 3.74]) and unsuccessful (baseline: 3.37, 95%CI [3.28; 3.47], follow-up: 3.49, 95%CI [3.39; 3.58]) registrants to Dry January [19]. Self-rated physical-health was greater at both 1-month and 6-month follow-up for successful registrants (baseline: 3.26, 95%CI [3.17; 3.35], 1-month follow-up: 3.47, 95%CI [3.39; 3.56], 6-month follow-up: 3.47, 95%CI [3.39; 3.56]) [19]. Participants to Dry July reported having changed their diet (2012: 36%, 2013: 22%), and having increased their current exercise program (2012: 36%, 2013: 22%) at the mid-year health check [25, 26]. 21% of 2019 Dry July participants reported feeling healthier [28].

Registrants reported other benefits of Dry January such as savings (63%), improved sleep (56%), more energy (52%), better health (50%), weight loss (38%). Improvement in all five of these domains was greater among successful participants (savings *d* = 0.13; *p* < 0.01, sleep *d* = 0.15; *p* < 0.01, energy *d* = 0.14; *p* < 0.01, health *d* = 0.12; *p* < 0.01, weight *d* = 0.24; *p* < 0.01) [18] (Table 2). The most commonly reported benefits during Febfast were also savings (52.2%), improved sleep (40.5%), weight loss (38.1%) and improved overall health (35.3%) [21]. 46.5% of Febfast registrants reported a reduction of their tobacco consumption during the event [21].

In the UK, odds of at-risk drinkers reporting a current attempt to restrict alcohol consumption was significantly higher in January compared to the other months (OR = 1.46, 95%CI [1.25;1.70]), as were the odds of at-risk drinkers citing Dry January as a motive in their most recent attempt to restrict consumption (OR = 2.29, 95%CI [1.62;3.22]) [22].

## Discussion

Each year, OMACs attract an increasing number of participants. For example, even if it still represents less than one percent of the Australian adult population in 2019, 44,000 people officially registered for Dry July [28], while they were 16,787 in 2016 and 9,532 in 2010 [34]. Regarding Dry January, 4,000 people participated in the 2014 campaign while they were 3.9 millions in 2020, that is, approximately 7.5% of the UK adult population [35, 36]. However, for ensuring the continued success of such campaigns, it is important to inform participants whether these programs meet harm reduction objectives. This review thus aimed to determine the profile of participants in the different national one-month abstinence campaigns, to estimate the rates and factors of success, and to explore the associated subjective benefits in participating in or completing the challenge.

Based on the studies pertaining to Dry January, it seems that those taking part in the challenge were more likely to be heavier drinkers, more concerned about their health, and had higher levels of incomes and education. The latter aspects are consistent with those reported elsewhere: the concern for healthy behaviors is more developed among individuals with higher education and incomes [37, 38]. However, this relationship is probably mediated, at least partially, by the overall level of education received, including during school years, suggesting that sustained and universal health education programs could help to bridge this gap [39]. The finding that females were more attracted in participating in abstinence campaigns is possibly in line with the fact that females are in general more concerned about health-related behaviors [40]. However, being a male led to better chance of successfully complete the abstinence campaign, specifically for campaigns promoting restriction of alcohol use. These results may reflect cultural differences across gender, with respect to alcohol use and alcohol-related representations [41].

Completing the one-month abstinence challenge was found to be associated with lower drinking patterns and better psychosocial functioning at baseline. Thus, it is interesting to note that those participating in the abstinence campaigns had more elevated drinking patterns compared to the non-participating alcohol users, whereas those achieving the challenge had lower drinking patterns compared to those who did not. Another important factor of success was the registration and active participation in social media communities. This is in line with the overall finding that interactive social media on the Internet can be a very effective tool to change health behaviors in the general population [42]. There may be some biases in this finding as participants who registered on social communities might be the most motivated ones, which could explain a better success in achieving the challenge. However, sharing the experience and the difficulties encountered during of a long time period of alcohol abstinence on a virtual community was designated as the most efficient strategy to successfully reach the abstinence goal during the online HSM program [7]. In this program, other strategies which were reported to be efficient to abstain from alcohol include the engagement in alcohol-free activities, the use of non-alcoholic beverages instead of alcohol, support from family and friends, and anticipation of social events [7]. On the contrary, anxiety, stress, negative emotions, social pressure to drink, loneliness, boredom, and no social support were reported as barriers to maintain alcohol abstinence [7]. Considering those dimensions as potential factors for success or failure in national one-month abstinence campaigns would be relevant in further studies.

Many participants in OMACs reported subjective improvements in health, including improved sleep, weight loss, an increased “energy”. An important finding is that Dry January participants also reported to have tried to increase their physical activity and to improve their diet, which was also reported by Dry July participants during the mid-year health check. This may suggest that these campaigns are actually not merely alcohol-focused for many participants, and might consist for them to a health-focused month, in particular when it is the first month of the year immediately after the end of year celebrations. This finding might have important implications for the evolution of the communication around these prevention campaigns. Moreover, improvement in health after one-month alcohol abstinence was objectively demonstrated for several parameters in a study with drinkers drinking above national guidelines where one-month alcohol abstinence led to a decrease in blood pressure, decrease in circulating concentrations of cancer-related growth factors, decrease in insulin resistance and weight reduction compared to the non-abstinent group [43].

Several limitations of this review should be addressed. Firstly, the majority of the available data comes from the UK Dry January initiative, which may restrain the generalizability of the conclusions. Secondly, there is a general paucity of studies for this kind of campaigns, and those available were non-randomized, but undertaking randomized studies in general prevention campaign is difficult, although some argue that it is feasible and that it should be done more often [44]. Moreover, we chose to focus only on the one-month abstinence campaigns, whereas other similar events involved a longer duration [6, 45]. However, we hypothesized that the responses to the questions addressed in this review were different for longer abstinence periods, including the profile of the participants, the rates and factors of success, and the subjective benefits of stopping alcohol. For instance, a 3-month abstinence campaign stands during the Buddhist-Lent period in Thailand, with factors being associated with successful alcohol abstinence including drinking frequency before the campaign but also religion and making a public commitment [45], and thus, parallels can hardly be made with these types of campaigns. Another limitation is that we only focused on the literature written in English and it’s therefore possible that we missed some data. As OMACs are far more recent in French speaking countries, we chose not to search for articles written in French language, assuming no data would yet be available. Moreover, if we had considered not English-only literature, it would have been methodology and deontologically required to explore all the languages concerned, including Dutch or Hungarian, which would have required additional skills and time for what we deemed as a probably limited result. Last, there was an important heterogeneity between the different national campaigns, their name, their goal including collecting funds for charities such as in Dry July, their overall organization, the place of social media, the chosen month for the campaign, and the local cultural differences regarding alcohol use and alcohol-related representations.

In order to increase the interest of the media and the general public in this kind of campaigns, it could be relevant to harmonize internationally the OMACs and to launch them simultaneously in neighboring countries. International discrepancies might persist though, at least between the two hemispheres, as these campaigns are generally set up during the winter. Furthermore, although individual factors of success and failure have been investigated in the UK Dry January challenge, barriers encountered and strategies implemented by participants to achieve success are yet to be identified, for instance the impact or use of societal factors including support by public authorities, as well as social support provided by relatives, social media or online communities as the ones existing in the HSM program. At last, a better understanding of the different profile of participants in the future may lead to improvement in communication strategies and digital tools of alcohol abstinence campaigns.

A remaining theoretical question is whether OMACs may be formally related to harm reduction policies. Originally, the harm reduction approach targeted people who used illicit drugs, and were more focused on reducing harms than promoting an overall reduction in the level of use [46]. However, in practice, harms are generally related to the frequency and average level of use and it is particularly relevant for alcohol, for which the indubitable related harms [47] are considered to be largely dose-related [48, 49]. Consequently, durably reducing the level of alcohol use is associated with a subsequent reduction in alcohol-related harms, either in populations with AUD, as well as in the general population [43, 50]. For these reasons, OMACs are generally considered as belonging to harm reduction policies [22, 51], even if they sensibly differ from the original meaning of the concept [52].

In conclusion, the present review identified some useful insights into OMACs, which offers a more comprehensive understanding of the effects of these interventions and of their harm reduction potential in the general population, but it also emphasizes the need for further researches to fill the remaining gaps about their objective benefits, the strategies deployed by the participants throughout the whole challenges, and the practical ways to reach an extended part of the population.

## Data Availability

Not applicable.
